# Factors Influencing Hospital Nurses' Workplace Bullying Experiences Focusing on Meritocracy Belief, Emotional Intelligence, and Organizational Culture: A Cross-Sectional Study

**DOI:** 10.1155/2024/1637066

**Published:** 2024-08-07

**Authors:** Insil Jang, Sun Joo Jang, Sun Ju Chang

**Affiliations:** ^1^ Department of Nursing Chung-Ang University, Seoul, Republic of Korea; ^2^ College of Nursing The Research Institute of Nursing Science Seoul National University, Seoul, Republic of Korea

## Abstract

**Aims:**

To identify the factors influencing hospital nurses' workplace bullying experiences (victim and perpetrator aspects) focusing on meritocracy beliefs, emotional intelligence, and organizational culture.

**Background:**

Workplace bullying remains a major issue in nursing despite decades of research and policy-making. Therefore, comprehensively understanding the individual and institutional factors affecting workplace bullying from both the victim and perpetrator perspectives is crucial.

**Methods:**

In October 2022, 379 nurses working in South Korean tertiary hospitals were surveyed using a self-reported online questionnaire. Meritocracy beliefs, emotional intelligence, workplace bullying experiences, and nursing organizational culture were measured using the Meritocracy Belief Scale, Wong and Law Emotional Intelligence Scale, Negative Acts Questionnaire-Revised, and Positive Nursing Organizational Culture Measurement Tool, respectively.

**Results:**

Gamma regression analysis revealed that, for workplace bullying, the factors influencing the victim aspect were the experience of witnessing bullying in the workplace, organizational culture, and meritocracy beliefs. In contrast, the factors affecting the perpetrator aspect were emotional intelligence, meritocracy beliefs, and experience of bullying at work.

**Conclusion:**

Decreasing nurses' degree of meritocratic hubris in a positive organizational culture and increasing their emotional intelligence are necessary to prevent and intervene in workplace bullying. *Implications for Nursing Management*. Targeted approaches are needed to address and mitigate the detrimental effects of factors influencing workplace bullying. These approaches could include interventions that improve nurses' emotional intelligence, assess their level of meritocracy beliefs, and offer opportunities for self-reflection on meritocratic hubris. Such initiatives may be necessary to effectively tackle workplace bullying and promote a healthier nursing work environment.

## 1. Introduction

Workplace bullying has remained an ongoing problem in nursing for the last four decades and threatens to persist in the future [1, 2]. It results in negative consequences for individual nurses, patients, and organizations worldwide [1, 3]. The factors that cause workplace bullying are diverse, and to understand workplace bullying in the nursing profession, integrating both individual disposition and institutional factors is necessary [4]. According to a cross-cultural scoping review [5] and a systematic review [6], the factors influencing workplace bullying among nurses are divided into two levels, individual and institutional. For individual-level antecedents of workplace bullying, demographics (gender, age, marital status, working experience, education level, etc.) and personality traits were reported. Institutional-level antecedents included work characteristics, such as work overload, staffing, working condition, leadership style, and organizational culture. These two types of factors are not mutually exclusive; thus, workplace bullying is often perceived as an outcome of interactions between these two factors [7]. In this study, as potential factors affecting workplace bullying, we focused on meritocracy and emotional intelligence at the individual level and organizational culture at the institutional level. Previous studies have mainly been limited to the victim aspects of workplace bullying, largely ignoring perpetrator aspects [2]. In the few studies on perpetrator aspects, in line with the incivility spiral effect [8], it was only found that the more workplace bullying was experienced, the higher reciprocated perpetration [9]. In this regard, identifying factors associated with workplace bullying victim and perpetrator aspects and creating intervention plans for both aspects are necessary in nursing management [2, 10]. This study integrates discussions from the perspectives of both victims and perpetrators regarding workplace bullying among nurses.

Meritocracy is a concept proposed by Young [11], referring to a fair and equitable system of gaining resources, status, and rewards based on individuals' intelligence, abilities, achievements, and efforts. In the nursing context, it refers to the idea of promoting and advancing nurses based on their skills, knowledge, and contributions to the field [12]. Contemporary youth highly value processes linked to ability [13]. The combination of meritocracy and fairness is known as “meritocratic justice,” which refers to the expectation of achieving fairness based on meritocracy [14]. The meritocratic justice is associated with the perception that acquiring resources and status based on ability or effort is justifiable [15]. Furthermore, meritocratic belief refers to a strong belief in the expectation of achieving fairness based on meritocracy. This belief can lead to a preference for intense competition and decreased sensitivity to inequality [15]. Consequently, meritocratic beliefs may cause nurses to attribute their success or desirable behavior to internal factors such as ability or effort, while blaming external factors such as luck or situational idiosyncrasies for their failures or undesirable behaviors. According to a longitudinal study [16], endorsing meritocratic beliefs during their youth has affected their own socioeconomic status across their life span. Such attributions can hinder mutual understanding and potentially contribute to workplace bullying among nurses. Moreover, meritocratic beliefs may lead to the exclusion or devaluation of individuals who do not fit traditional merit-based criteria [17]. This can be explored in the nursing context by examining how meritocratic ideals inadvertently perpetuate inequality or hinder diversity and inclusion in the profession. Based on these backgrounds, we hypothesized that stronger meritocracy belief can influence workplace bullying experiences from the perspectives of both victim and perpetrator (hypothesis 1).

Emotional intelligence encompasses the ability to monitor and comprehend one's own emotions, as well as those of others, and utilize this information to guide decision-making and actions [18]. In healthcare professions, high levels of emotional intelligence have positive effects on compassionate care and nursing performance [19, 20]. Extensive research has established a correlation between nurses' emotional intelligence and important aspects of their practice, including person-centered care [21], nursing productivity [22], and nursing care quality [23]. Additionally, emotional intelligence contributes positively to the performance of caring roles among nurses in multispecialty hospital settings [24]. Notably, emotional intelligence enables the effective management of both patients' needs and demands within work relationships, leading to increased collaboration among nurses [18, 25]. Nurses with a low profile of emotional intelligence may become perpetrators of workplace bullying [10]. Nurses themselves acknowledge the importance of emotional intelligence as a quality that underpins their effectiveness as healthcare professionals and leaders, aids the understanding of others' needs and concerns, and facilitates individualized care [26]. Consequently, emotional intelligence can mitigate or prevent workplace bullying by promoting an understanding of one's own emotions, as well as the emotional dynamics among fellow nurses. We hypothesized that lower emotional intelligence significantly contributes to workplace bullying experiences from the perspectives of both victim and perpetrator (hypothesis 2).

Nursing organizational culture refers to the shared values, beliefs, and behaviors among nurses within an organization that are acquired by and transmitted to colleagues and significantly shape thoughts and actions [27, 28]. It has been identified as a crucial organizational factor that influences workplace bullying [29, 30]. Nurses who are bullied may passively accept or endure bullying because of the belief that reporting it will be ineffective, thereby highlighting the cyclical nature of bullying fostered by ingrained organizational cultures [31]. Different types of organizational cultures—such as those that are relationship-oriented, hierarchical-oriented, performance-oriented, and innovation-oriented—have significant effects on workplace bullying [29, 32, 33]. Positive organizational culture among tertiary hospital nurses was associated negatively with the workplace bullying victim aspect [10]. Thus, we hypothesized that a less positive organizational culture can influence workplace bullying experiences from both victim and perpetrator perspectives (hypothesis 3).

Addressing bullying is crucial given its prevalence and impact on unhealthy work environments [5]. In addition, with the increasing use of social media platforms and internal communication systems in hospitals, both face-to-face bullying and cyberbullying have become more prevalent [28]. According to a systematic review on interventions for nurses' workplace bullying [34], all the interventions enhancing communication skills, interpersonal relationships, assertiveness, trust, and empowerment were effective. These strategies enhancing self-reflection and empathy can be introduced to solve interpersonal conflicts and workplace bullying [34, 35], and are in line with developing effective interventions identifying meritocracy and emotional intelligence as factors affecting workplace bullying is crucial. Furthermore, the goal was to gain insights into the dynamics of workplace bullying in terms of victim and perpetrator aspects and contribute to the development of effective intervention strategies for creating healthier and more supportive nursing environments.

## 2. Materials and Methods

### 2.1. Study Design

A cross-sectional survey was conducted in accordance with the STROBE guidelines [35].

### 2.2. Participants and Data Collection

The inclusion criteria were as follows: (1) nurses working at tertiary hospitals in South Korea, (2) incumbent nurses providing direct and indirect care, and (3) nurses belonging to the nursing department. The exclusion criteria were as follows: (1) nurses working at secondary- or lower-level hospitals, and (2) newly graduated nurses in their probationary period (less than three months) considering that nurses are under special protection, supervision, and guidance of the nurse manager. Healthcare professionals in tertiary care settings are required to be highly specialized and experienced [36, 37]. They are exposed to risks of high levels of work stress and burnout [38] and are prone to experience workplace bullying. Considering known antecedents of workplace bullying among nurses [5], we have limited the institution level to tertiary hospitals. This study was conducted through an online survey using a link; there was no restriction on participants' geographical location (including all regions of South Korea).

To determine the required sample size for multiple regression analysis, the researchers utilized the software G^*∗*^Power 3.1.9.2, with a significance level of 0.05, a power (1–*β*) of 0.80, 13 predictors, and an effect size (*f*^2^) of 0.06 [10]. The calculated minimum sample size was 309. Considering a potential dropout rate of approximately 10% based on a previous online research study [39], the researchers posted an online survey link on the online community for nurses most frequently visited as an open call invitation. A warning was displayed to the participants not to respond more than once. Data collection occurred between October 27 and November 1, 2022, with a target of 345 participants. Although 440 responses were collected, the analysis included 379 participants after excluding 45 who were not workers at a tertiary hospital and 16 who were not belonging to the nursing department. All respondents received a coffee voucher after completing the online survey (worth $5).

### 2.3. Ethical Considerations

After receiving approval from the institutional review board of the authors' university (approval number: blinded for review, granted on September 7, 2022), the study was conducted in accordance with the guidelines outlined in the Declaration of Helsinki. To ensure ethical considerations, the research participants were provided with a comprehensive explanation detailing the research purpose, methodology, assurance of anonymity, option to withdraw from participation at any point if desired, and commitment to destroy all related information upon completion of the study. Following the provision of this information and electronic informed consent, the participants completed a questionnaire as part of the research process.

### 2.4. Instruments

Data on participants' sociodemographic (sex, age, marital status, religion, education level, and subjective health status) and work-related characteristics (total working years, current working unit, position, workplace bullying prevention training, and experience of workplace bullying) were obtained using a self-report questionnaire specifically developed for this study by the researchers. Validated instruments were used to assess meritocracy, emotional intelligence, organizational culture, and workplace bullying (victim and perpetrator aspects). Permission to use the Korean versions of these instruments was obtained from the original researchers and authors.

#### 2.4.1. Meritocracy Belief

The Meritocracy Belief Scale [14] was used to measure belief in meritocratic justice. It consists of eight questions on a 6-point Likert-type scale (1 = not experienced at all and 6 = very strongly experienced), and the scores range from 8 to 48 points. In the original study, the tool's convergent, discriminant, and content validity were verified, and Cronbach's *α* = 0.83 [15]. In this study, Cronbach's *α* = 0.79.

#### 2.4.2. Emotional Intelligence

Emotional intelligence was assessed using the Wong and Law Emotional Intelligence Scale, originally developed by Wong and Law [40] and adapted and validated in the Korean language by Jeong et al. [41]. This scale consists of 16 items in the following subdomains: self-emotional appraisal, others' emotional appraisal, regulation of emotion, and use of emotion. Each item is rated on a 7-point Likert scale, and the total score ranges from 16 to 112 points, with higher scores indicating higher levels of emotional intelligence. The internal consistency reliability of the original scale was reported as Cronbach's *α* = 0.76 to 0.89 [40], whereas in the Korean version, Cronbach's *α* = 0.88 (0.80 to 0.89) [41]. In this study, Cronbach's *α* = 0.90 (0.80 to 0.88).

#### 2.4.3. Organizational Culture

The Positive Nursing Organizational Culture Measurement Tool [42] was used to assess organizational culture. It comprises 26 items divided into the following subfactors: positive leadership of the nursing unit manager, pursuit of common values, formation of organizational relationships based on trust, and a fair management system. Each item is rated on a 5-point Likert scale ranging from 1 (not at all) to 5 (very much so). The total score ranges from 24 to 120 points, with higher scores indicating a stronger recognition of the positive aspects of the nursing organizational culture. The internal consistency reliability of the scale in the original study was Cronbach's *α* = 0.95 (0.83 to 0.95) [42]. In this study, Cronbach's *α* = 0.96.

#### 2.4.4. Workplace Bullying Experiences

The Korean version [43] of The Negative Acts Questionnaire-Revised (NAQ-R) [44] was used to assess workplace bullying experiences from the victim's perspective. The NAQ-R defines victims of workplace bullying as those who experienced direct and indirect aspects of work-related bullying, person-related bullying, or physical intimidation [44]. The NAQ-R consists of 22 items (i.e., being humiliated or ridiculed in connection with your work and having allegations made against you) rated on a 5-point Likert scale. The total score ranges from 22 to 110 points, with higher scores indicating more severe workplace bullying. The internal consistency reliability of the original scale was reported as Cronbach's *α* = 0.93 [44], while in the Korean version, Cronbach's *α* = 0.93 [43]. In this study, Cronbach's *α* = 0.96.

The Negative Acts Questionnaire-Perpetrator (NAQ-R-P) [45; currently under publication] was used to measure workplace bullying experiences from the perpetrator's perspective. The items of the NAQ-R-P were constructed by modifying items from the NAQ-R [44] that focused on victim aspects, adjusting them to situations involving perpetrators. The NAQ-R-P consists of 22 items rated on a 5-point Likert scale, with a scoring range of 22–110; higher scores indicate more severe workplace bullying instigation [44]. This scale is unidimensional, and its validity has been verified [45]. The Tucker–Lewis Index = 0.86, Incremental Fit Index = 0.90, Comparative Fit Index = 0.88, Normal Fit Index = 0.85, and Content Validity Index for the universal scale is 0.83 [45]. In the original study, Cronbach's ⍺ for the NAQ-R-P was 0.97 [45]. In this study, Cronbach's ⍺ was 0.97.

### 2.5. Data Analysis

The data were analyzed using IBM SPSS for Windows (version 26.0; IBM Corp., Armonk, NY, USA). Repeat participation in the survey link was strictly limited, and there were no missing values, as all questions were answered. Descriptive statistics were used to analyze the participants' sociodemographic and work-related characteristics, meritocracy, emotional intelligence, organizational culture, and workplace bullying. The normality of the variables was evaluated using the Shapiro–Wilk test. The normality assumption was not satisfied. Mann–Whitney U and Kruskal–Wallis tests were used to examine differences in workplace bullying (victim and perpetrator aspects) based on participants' sociodemographic characteristics. Spearman's rank correlation coefficient was used to assess the association among the major variables. Gamma generalized linear regression analysis was performed to determine the effects of the major variables on workplace bullying experiences (victim and perpetrator aspects).

## 3. Results

### 3.1. Participants' Sociodemographic and Work-Related Characteristics

Table 1 presents the sociodemographic and work-related characteristics of the participants. The participants' median age was 30.00 years (interquartile range: 27.00∼37.00, minimum: 22.00, maximum: 55.00), and the majority were women, accounting for 352 (92.9%) participants in the total sample. Among the participants, 231 (60.9%) were unmarried, and 222 (58.6%) were nonreligious. Regarding education, 280 (73.9%) participants held a bachelor's degree. In terms of occupation, 307 (81.0%) participants were registered staff nurses. The majority of participants (202, 53.3%) were currently working in general wards. The median of clinical experience was 6.00 years (interquartile range: 3.00∼11.00). Furthermore, 230 (60.7%) reported completing workplace bullying prevention training in the past year; 88 (23.2%) reported experiencing workplace bullying in the past year; 25 (6.6%) reported being workplace bullying perpetrators; and 149 (39.3%) reported witnessing their colleagues being bullied.

### 3.2. Differences in Victim and Perpetrator Aspects of Workplace Bullying by Sociodemographic and Work-Related Characteristics

The results of the analysis of the differences in workplace bullying (victim and perpetrator aspects) based on sociodemographic and work-related characteristics are presented in Tables 2 and 3, respectively.  Table 2 shows that workplace bullying (victim aspect) was significantly higher among participants who reported their subjective health status as “not healthy,” those who had experienced workplace bullying in the past year, and those who had witnessed workplace bullying in the past year. Similarly, Table 3 illustrates that workplace bullying (perpetrator aspects) was significantly higher among those who reported their subjective health status as “not healthy,” those who had been victims of workplace bullying in the past year, and those who had witnessed workplace bullying in the past year.

### 3.3. Correlation among the Major Variables

Workplace bullying experiences (victim aspect) were positively correlated with meritocracy beliefs (*ρ* = 0.13, *p*=0.010) and negatively correlated with emotional intelligence (*ρ* = −0.10, *p*=0.046) and organizational culture (*ρ* = −0.30, *p* < 0.001). Workplace bullying experiences (perpetrator aspect) were positively correlated with meritocracy beliefs (*ρ* = 0.16, *p*=0.002) and negatively correlated with emotional intelligence (*ρ* = −0.17, *p* < 0.001) and organizational culture (*ρ* = −0.19, *p* < 0.001). A significant positive correlation was found between the victim and perpetrator aspects of workplace bullying experiences (*ρ* = 0.50, *p* < 0.001) (Table 4).

### 3.4. Factors Affecting Workplace Bullying Experiences (Victim and Perpetrator Aspects)

#### 3.4.1. Victim Aspect of Workplace Bullying Experiences

The variables that were significant at *p* < 0.05 in the analysis of differences in workplace bullying (victim aspect) based on participants' characteristics, as well as the variables that were correlated with the victim aspect, were included in the Gamma generalized linear regression model. Factors influencing the victim aspect of workplace bullying were witnessing workplace bullying, meritocracy beliefs, and organizational culture (Table 5). From the perspective of victim, hypotheses 1 and 3 were supported; however, hypothesis 2 was not.

#### 3.4.2. Perpetrator Aspect of Workplace Bullying Experiences

The variables that were significant at *p* < 0.05 in the analysis of differences in workplace bullying (perpetrator aspect) based on participants' characteristics, as well as the variables that were correlated with the perpetrator aspect, were included in the Gamma generalized linear regression model. Factors influencing the perpetrator aspect of workplace bullying were workplace bullying experiences, meritocracy beliefs, and emotional intelligence (Table 6). From the perspective of perpetrator, hypotheses 1 and 2 were supported; however, hypothesis 3 was not.

## 4. Discussion

This study aimed to investigate the factors influencing workplace bullying experiences from both victims' and perpetrators' perspectives, focusing specifically on meritocracy beliefs, emotional intelligence, and organizational culture. There has been sustained interest in developing and implementing various intervention programs and solutions aimed at preventing workplace bullying, which persists as a significant issue within organizational culture [5, 32, 33]. Nevertheless, this study shows that efforts to eradicate workplace bullying have not bridged the gap in the perception of victims and perpetrators, indicating a significant lack of awareness among perpetrators. Moreover, as the number of witnesses to workplace bullying exceeds the number of victims, those who experience workplace bullying often perceive it as a by-product of organizational culture rather than fully recognizing the seriousness of the harm they have suffered [30, 31]. Previous research has highlighted the critical role of nursing organizational culture in influencing workplace bullying, emphasizing the necessity of implementing specific strategies and organizational-level interventions to avoid a hierarchy-oriented culture [27, 29–31]. These studies demonstrate that nurses who have been victims of workplace bullying often find it challenging to actively address the issue, with such repetitions frequently stemming from and reinforcing the characteristics of the organizational culture [29, 30].

The findings of this study revealed that several factors influenced the victim aspect of workplace bullying. Specifically, the experience of witnessing workplace bullying, meritocracy beliefs, and organizational culture emerged as significant factors associated with the occurrence of workplace bullying as a victim. Establishing an organizational culture rather than focusing solely on individual abilities or differences is crucial. Organizational initiatives aimed at cultivating an optimal workplace environment for nurses hold significance in enhancing nursing organizational culture [28, 46]. To date, various programs have been implemented at both individual and organizational levels to reduce the incidence of workplace bullying. For individual strengthening, initiatives such as depression and stress management and psychiatric counseling have been explored. On the organizational level, efforts have included participation in conflict management training programs, policies to facilitate smooth communication, and the application of social media within the hospital environment [34, 47, 48]. Both approaches must be easily accessible to the target audience, and integrating these initiatives into the organizational culture remains a future challenge.

Younger generations place a high value on meritocracy, and their stronger meritocracy beliefs can lead to uncertain outcomes and contribute to workplace bullying toward others [14, 15, 17]. Relying solely on quantitative evaluation without relational assessment can also contribute to workplace bullying [32]. Therefore, incorporating qualitative evaluations alongside quantitative measures is necessary for a comprehensive understanding of individuals. For this reason, peer evaluation systems have been established in hospitals to maintain an appropriate level of meritocracy [49]. In addition, it is essential to employ various strategies to promote teamwork, beginning with fostering a relationship-oriented nursing organizational culture that emphasizes mutual respect and prevents careless treatment among health professionals [17, 28, 47]. Understanding the occurrence of workplace bullying, especially among colleagues, is intricately linked to the complexities of human behavior, and employing a naturalistic approach to inquiry can provide deeper insights into this phenomenon [50]. Efforts should be made to promote the significance of processes and relationships alongside tangible outcomes through a horizontal organizational culture and transparent communication channels.

This study identified several factors that influence the perpetrator aspects of workplace bullying. These factors included experience of workplace bullying as a victim, meritocracy beliefs, and emotional intelligence. This study underscores the significance of implementing strategies aimed at enhancing emotional intelligence as essential measures to prevent and mitigate future workplace harassment. Lu and Shorey found that nurses were aware of the importance of emotional intelligence in clinical practice, demonstrated a strong interest in improving their emotional intelligence skills, and recognized potential obstacles to the development of emotional intelligence [26]. Improving emotional intelligence not only enhances the value of patient-centered, personalized nursing care but also fosters stronger relationships among nurse colleagues. Emotional intelligence is acknowledged as a valuable asset enhancing performance and fostering effective group cohesion among nurses, serving as a universal buffer and coping strategy for work-related stress [51]. However, despite recognizing the potential for improvement through learning and training, there remains a scarcity of emotional intelligence promotion and intervention programs specifically tailored to healthcare professionals [52]. Hence, we recommend that more nursing leadership and intervention studies be conducted to ascertain the role of enhanced emotional intelligence among nurses. In particular, efforts to strengthen emotional intelligence include managers incorporating emotional intelligence competencies as a key criterion in the recruitment strategy for new nurses and university professors enhancing educational curricula to better develop these competencies [51, 52]. It is imperative for nurse managers to adeptly understand and harness emotional intelligence competencies.

Among the factors influencing workplace bullying by perpetrators, the concept of meritocracy warrants a balanced discussion due to its potential for both positive and negative effects. If achievements are the sole focus, the prevalence of meritocracy beliefs may lead to justifying both workplace bullying victimization and perpetration [15]. Balancing meritocracy beliefs to account for both nurses' care outcomes and their impact on patients' quality of life is essential for fostering a healthy and supportive workplace environment. Additionally, based on the results of this study, which identified the experience as a victim of workplace bullying as an influencing factor for the perpetrator, it supports existing research findings that victims change into perpetrators over time [53, 54]. It is essential to make individual or organizational efforts to avoid becoming a victim of workplace bullying and establishing an organizational culture for this is important.

### 4.1. Limitations

In the current study, data were collected through an online survey as an open call invitation and institutions could not be determined; therefore, it may have led to self-selection bias and institutional effects cannot be identified. In addition, organizational factors such as nurse staffing or other work environments were not investigated. In future research, employing a random multistage sampling is recommended, and institutional characteristic effects should be assessed through multilevel analysis. To understand the situation of the nursing field, the status of meritocracy beliefs, emotional intelligence, organizational culture, and workplace bullying experiences among the various clinical settings should be compared. Additionally, the use of only self-reported measures suggests the need to include objective indicators, such as observations of workplace bullying behavior, for a more comprehensive analysis. Furthermore, to increase the explanatory power of the influencing factors of victims and perpetrators, we must pay attention to relationships through mixed studies. Future research should explore the relationship between emotional intelligence, meritocratic beliefs, and organizational culture. Despite these limitations, this study provides valuable insights into the factors influencing workplace bullying among clinical nurses by examining the perspectives of both victims and perpetrators.

## 5. Conclusions

In nursing management, customized approaches are crucial for addressing the factors contributing to workplace bullying and mitigating its negative consequences. The findings highlight the importance of targeted interventions, such as evaluating the impact of meritocracy beliefs, fostering self-reflection on potential meritocratic hubris, and enhancing nurses' emotional intelligence. Establishing an organizational culture that prioritizes relationships, trust, and open communication rather than solely emphasizing individual abilities or differences is paramount. To prevent workplace bullying among nurses, the implementation of protocols to prevent disruptive behaviors and foster teamwork is essential. These initiatives are critical in effectively combating workplace bullying and creating healthier and more supportive work environments for nurses.

## Figures and Tables

**Table 1:** General and work-related characteristics of the participants (*N* = 379).

Characteristics	Categories	*N* (%)	Median (interquartile range)
Age (years)			30.00 (27.00∼37.00)

Gender	Female	352 (92.9)	
	Male	27 (7.1)	

Marital status	Single	231 (60.9)	
	Married	148 (39.1)	

Religion	No	222 (58.6)	
	Yes	157 (41.4)	

Educational level	3-year college	48 (12.7)	
	Bachelor's degree	280 (73.9)	
	Master's degree or higher	51 (13.5)	

Subjective health status	Not healthy	228 (61.2)	
	Healthy	151 (39.8)	

Career length (years)			6.00 (3.00∼11.00)

Current working unit	Intensive care unit	81 (21.4)	
	Ward	202 (53.3)	
	Outpatient department	46 (12.1)	
	Others^†^	50 (13.2)	

Position	Staff nurse	307 (81.0)	
	Charge nurse or above	72 (19.0)	

Workplace bullying prevention training^‡^	No	149 (39.3)	
	Yes	230 (60.7)	

Experience of workplace bullying as victims^‡^	No	291 (76.8)	
	Yes	88 (23.2)	

Experience of workplace bullying as perpetrators^‡^	No	354 (93.4)	
	Yes	25 (6.6)	

Witnessed their colleagues being bullied^‡^	No	230 (60.7)	
	Yes	149 (39.3)	

*Notes*. ^†^Emergency department, operating room, and clinical specialist, and ^‡^within one year.

**Table 2:** Comparison of differences in workplace bullying (victim aspect) by general and work-related characteristics (*N* = 379).

Characteristics	Categories	*N*	Workplace bullying (victim aspect)		
			Mean rank	*U* or *H*	*p*
Gender	Female	352	188.52	0.95	0.342
	Male	27	209.31		

Marital status	Single	231	194.82	1.07	0.284
	Married	148	182.48		

Religion	No	222	192.17	0.46	0.647
	Yes	157	186.94		

Education level	3-year college	48	202.57	0.84	0.659
	Bachelor's degree	280	189.03		
	Master's degree or higher	51	183.49		

Subjective health status	Not healthy	228	205.54	3.39	<0.001
	Healthy	151	166.53		

Current working unit	Intensive care unit	81	191.56	5.25	0.155
	Ward	202	198.55		
	Outpatient department	46	158.92		
	Others^†^	50	181.54		

Position	Staff nurse	307	190.81	3.06	0.765
	Charge nurse or above	72	186.53		

Workplace bullying prevention training^‡^	No	149	184.05	0.85	0.395
	Yes	230	193.85		

Experience of workplace bullying as perpetrators^‡^	No	354	186.90	2.08	0.038
	Yes	25	233.96		

Witnessed their colleagues being bullied^‡^	No	230	155.86	7.54	<0.001
	Yes	149	242.70		

*Notes*. Others^†^: emergency department, operating room, and clinical specialist, and ^‡^within one year.

**Table 3:** Comparison of differences in workplace bullying (perpetrator aspect) by general and work-related characteristics (*N* = 379).

Characteristics	Categories	*N*	Workplace bullying (perpetrator aspect)		
			Mean rank	*U* or *H*	*p*
Gender	Female	352	187.04	1.91	0.057
	Male	27	228.65		

Marital status	Single	231	193.10	0.69	0.491
	Married	148	185.17		

Religion	No	222	187.21	0.59	0.555
	Yes	157	193.95		

Education level	3-year college	48	187.42	0.47	0.792
	Bachelor's degree	280	192.07		
	Master's degree or higher	51	181.08		

Subjective health status	Not healthy	228	205.00	3.28	0.001
	Healthy	151	167.35		

Current working unit	Intensive care unit	81	183.87	7.14	0.068
	Ward	202	199.56		
	Outpatient department	46	153.14		
	Others^†^	50	195.23		

Position	Staff nurse	307	193.34	1.67	0.220
	Charge nurse or above	72	175.78		

Workplace bullying prevention training^‡^	No	149	197.58	1.09	0.278
	Yes	230	185.09		

Experience of workplace bullying as victims^‡^	No	291	175.55	4.68	<0.001
	Yes	88	237.78		

Witnessed their colleagues being bullied^‡^	No	230	170.54	4.30	<0.001
	Yes	149	220.04		

*Notes*. Others^†^: emergency department, operating room, and clinical specialist, and ^‡^within one year.

**Table 4:** Correlations among variables (*N* = 379).

	Career length	Meritocracy belief	Emotional intelligence	Organizational culture	Workplace bullying (victim)	Workplace bullying (perpetrator)
	*r* (*p*)					
Career length	1					
Meritocracy belief	0.06 (0.266)	1				
Emotional intelligence	0.06 (0.234)	0.29 (<0.001)	1			
Organizational culture	−0.14 (0.008)	0.23 (<0.001)	0.48 (<0.001)	1		
Workplace bullying (victim)	−0.08 (0.105)	0.13 (0.010)	−0.10 (0.046)	−0.30 (<0.001)	1	
Workplace bullying (perpetrator)	0.00 (0.959)	0.16 (0.002)	−0.17 (<0.001)	−0.19 (0.001)	0.50 (<0.001)	1

**Table 5:** Factors affecting workplace bullying (victim aspect) (*N* = 379).

Variables	*B*	SE	Wald	*p*	95% Wald CI	
					Lower	Upper
(Constant)	4.14	0.11	1305.71	<0.001	3.91	4.26
Subjective health status^†^	−0.02	0.04	0.38	0.540	−0.10	0.05
Experience of workplace bullying perpetrators^†^	−0.02	0.07	0.08	0.778	−0.15	0.11
Witnessed their colleagues being bullied^†^	0.24	0.04	47.41	<0.001	0.17	0.31
Meritocracy belief	0.01	0.00	9.24	0.002	0.01	0.02
Emotional intelligence	0.01	0.00	0.71	0.401	−0.01	0.01
Organizational culture	−0.01	0.00	39.40	<0.001	−0.01	−0.01
*χ* ^2^ = 105.85, *p* < 0.001, log likelihood = −1609.91, deviance/df = 0.11						

*Notes*. SE, standard error; VIF, variance inflation factor; CI, confidence interval. ^†^Dummy variable (reference): subjective health status (not healthy), experience of workplace bullying perpetrators (no), and witnessed their colleagues being bullied (no).

**Table 6:** Factors affecting workplace bullying (perpetrator aspect) (*N* = 379).

Variables	*B*	SE	Wald	*p*	95% Wald CI	
					Lower	Upper
(Constant)	3.89	0.12	972.97	<0.001	3.64	4.13
Gender^†^	0.13	0.07	3.32	0.069	−0.01	0.26
Subjective health status^†^	−0.01	0.04	0.01	0.947	−0.08	0.08
Experience of workplace bullying as victims	0.13	0.05	6.59	0.010	0.03	0.23
Witnessed their colleagues being bullied	0.08	0.05	2.88	0.089	−0.01	0.17
Meritocracy beliefs	0.02	0.00	41.87	<0.001	0.01	0.02
Emotional intelligence	−0.01	0.00	24.26	<0.001	−0.02	−0.01
Organizational culture	−0.01	0.00	1.73	0.189	−0.01′	0.01
*χ* ^2^ = 98.94, *p* < 0.001, log likelihood = −1485.97, deviance/df = 0.12						

^†^Dummy variable (reference): gender (female), subjective health status (not healthy), experience of workplace bullying victims (no), and witnessed their colleagues being bullied (no). SE, standard error; VIF, variance inflation factor; CI, confidence interval.

## Data Availability

The data used to support the findings of this study are available from the corresponding author upon request.
